# Supporting Primary Care for Medically and Socially Complex Patients in Medicaid Managed Care

**DOI:** 10.1001/jamanetworkopen.2024.58170

**Published:** 2025-02-03

**Authors:** Arlene S. Ash, Matthew J. Alcusky, Randall P. Ellis, Meagan J. Sabatino, Frances E. Eanet, Eric O. Mick

**Affiliations:** 1 Department of Population and Quantitative Health Sciences, UMass Chan Medical School, Worcester, Massachusetts; 2 Department of Economics, Boston University, Boston, Massachusetts

## Abstract

**Importance:**

In 2023, the Massachusetts Medicaid and Children’s Health Insurance Program (MassHealth) required accountable care organizations (ACOs) to increase payments to primary care practices and shift to monthly payments, currently calibrated to historical revenues and enhanced practice capabilities, such as being staffed to address behavioral health needs. To prevent rewarding practices for avoiding difficult patients, future payments to primary care practices should reflect their patients’ apparent need.

**Objective:**

To describe MassHealth’s initiative and a complexity-adjusted payment model.

**Design, Setting, and Participants:**

This cross-sectional study of payment model development and performance was conducted between February 2022 and November 2024. Participants included all 2019 Massachusetts Medicaid managed-care eligible members who were enrolled for 183 days or longer.

**Exposures:**

Medical and social complexity.

**Main Outcomes and Measures:**

For each member, the primary care activity level (PCAL) outcome proxies the resources that primary care clinicians need to provide comprehensive, coordinated care. Models were evaluated via *R*^2^ and through ratios of observed-to-expected (ie, estimated by the model) outcomes for selected subgroups, which will be approximately 1.0 when payments and expected costs are well matched. The implications of paying practices using PCAL (vs a model based only on age and sex) were explored by examining financial and practice-level characteristics in high and low deciles of practice-level estimated mean.

**Results:**

Among 1 092 742 MassHealth members enrolled in 3602 primary care practices (1 014 252 person-years; mean [SD] age, 25.9 [18.4] years; 538 065 [53.1%] female), the PCAL model achieved *R*^2^ = 69.6% and estimates within 10% of observed PCAL spending for high-risk populations (mental health disorders, substance use disorders, complex chronic conditions, and disabilities) and across racial and ethnic groups. Age-adjusted and sex-adjusted payments would overpay practices in the lowest-need decile by 10% and underpay those in the highest-need decile by 34%, while the PCAL model would match payment to estimated need almost exactly in the lowest decile and underpay by just 6% in the highest decile.

**Conclusions and Relevance:**

MassHealth’s 2023 reform invests in primary care. This cross-sectional study developed a risk model that can adjust primary care payments to patient needs. Neither age and sex adjustments nor inflated historical payments would provide adequate resources to primary care practices caring for the most complex patients.

## Key Points

**Question:**

Can models be used to allocate prospective payments to primary care practices in a way that supports caring for medically and socially complex patients?

**Findings:**

In this cross-sectional study using data from 1.1 million MassHealth members enrolled in 3602 primary care practices, a primary care activity level model achieved *R*^2^ of 69.6% and estimates within 10% of an observed measure of primary care spending need for high-risk populations and across racial and ethnic groups.

**Meaning:**

This study shows how patient complexity can be used to allocate capitated payments among primary care practices.

## Teaser

This cross-sectional study examines the Massachusetts Medicaid and Children’s Health Insurance Program 2023 initiative to require accountable care organizations to increase payments to primary care practices and shift to monthly payments.

## Introduction

Primary care clinicians are ideally situated to provide care continuity, manage chronic disease, lengthen healthy lives, and ensure health equity.^1,2,3^ Yet, primary care in the US is increasingly inaccessible^4^ owing to the predominant fee-for-service payment model with lower payments for primary vs specialist care and inadequate ways to bill for the complexity of coordinating care for people with multiple medical and social problems.^5^

Since 2018, the Massachusetts Medicaid and Children’s Health Insurance Program (MassHealth) has sought to promote coordinated care through accountable care organizations (ACOs).^6^ MassHealth has 2 types of ACOs: (1) primary care ACOs that directly contract with MassHealth and (2) accountable care partnership plans where MassHealth pays a managed care organization using capitation. Before 2023, primary care ACOs paid their clinicians via fee-for-service, and although managed care organizations could choose how they paid primary care practitioners in their partnering ACOs, in practice, fee-for-service predominated. In April 2023, MassHealth increased its focus on primary care, requiring that ACOs (1) increase the total dollars paid to primary care practices, (2) pay practices to care for their enrolled patient panels via prospectively determined monthly payments unlinked from the specific services they provide, (3) increase baseline expectations for the clinical criteria all practices must meet, and (4) make larger payments for practices that provide expanded access and services, especially for behavioral health (eTable 1 in Supplement 1). Current practice payments are based on augmented historical payments and service-level tier.^7,8,9^

Although initially basing prospective payments on inflated historical costs is reasonable, monthly payments must ultimately reflect current patient needs; failing to recalibrate the model rewards practices for avoiding complex patients. Thus, MassHealth asked our research team at UMass Chan to develop a payment model to address differences in patient risk, including equity concerns such as potential underservice in low-income neighborhoods and the greater resources needed to care for people without secure housing. There is a rich literature on the importance of addressing social risk in alternative payment initiatives and the role of area-based measures for this purpose.^10,11,12^

The federal government has used morbidity-based risk adjustment to allocate payments to health plans for the expected total cost of care since 2000.^13,14^ Various initiatives have expanded on this idea to account for differences in social risk as well, directing more money to plans or practitioners who serve members living in neighborhoods with fewer resources.^15,16,17^ The development of federal methods for paying health plans parallels what we propose for paying primary care practices: (1) construct the cost outcome of interest on a large, representative database with rich information on the characteristics of individual members; (2) build a model to estimate that outcome, checking to ensure that cost estimates for groups of patients with difficult-to-treat problems are close to their observed costs; and (3) use model-estimated costs to allocate primary care payments among health plans in a budget-neutral way.

The risk model we developed for MassHealth’s primary care reform initiative is somewhat different in that it is designed to allocate payments among primary care practices in a subcapitation arrangement financed from each plan’s global budget. Another difference is that the fee-for-service bills in the data we use for modeling fail to capture many primary care patient management activities. Our solution is to use MassHealth data to create (and then estimate) a proxy outcome, the primary care activity level (PCAL), for the enhanced primary care resources needed.

The PCAL framework was developed by Ash et al^18^ for allocating a risk-adjusted bundled payment among commercially insured primary care practices. The motivating idea is that the kinds of patients who tend to use substantial non–primary-care services should benefit from more primary care services, both to coordinate and manage that care and to avoid exacerbations of chronic diseases that generate acute service use. Importantly, we do not pay more for patients when they use more services, but rather when their medical and social characteristics are associated with higher expected use.

## Methods

### Study Setting

We used MassHealth data (enrollment, eligibility, practitioner, claims, and encounter records) to study its entire managed care–eligible population (members younger than 65 years for whom MassHealth was the primary insurer), so long as they were enrolled for at least half of 2019, the most recent available data not affected by COVID-19. Most enrollees (658 923 of 1 092 742 individuals [60.3%]) were present all year; the half-year enrollment requirement removed only 14.6% of person-years, and the mean (SD) enrollment duration was 11.33 (1.55) months. We did not account for gaps in coverage, which were extremely rare. MassHealth is working to improve the completeness of the reported racial and ethnic information that ACOs collect by various methods. Although over 40% of our study population had unknown race and ethnicity, we used the nonmissing data to explore payment equity. As an operations project, this study was considered not human participants research by UMass Chan’s institutional review board; thus, informed consent was not needed in accordance with 45 CFR §46. Analyses were conducted between February 2022 and November 2024, and this report follows the Strengthening the Reporting of Observational Studies in Epidemiology (STROBE) reporting guidelines for cross-sectional studies.

### Constructing the PCAL Outcome

We defined the PCAL outcome as the sum of differing percentages of costs from 5 categories: primary care (100%), specialty care (6%), inpatient hospital care (6%), emergency department (30%), and pharmacy (9%). The rationale is that primary care practices should devote resources to deliver and coordinate care for patients who are likely to be, for example, visiting specialists for consultations or treatment, taking prescribed drugs, hospitalized, or admitted to emergency departments. The fractions used were originally determined^18^ to align with a panel of primary care physicians’ estimates of how much of their time was spent doing things like managing prescriptions, following up with hospitalized patients, and so forth; they were lightly modified for MassHealth (eTable 2 in Supplement 1). Note that because the model is only used to create a practice-specific multiplier (mean PCAL for a practice’s patients divided by population mean PCAL) to allocate a predetermined total budget among practices, practice-level allocations are not highly sensitive to the precise percentages of non–primary care service types used in constructing PCAL.

For each person and cost category, we capped values at the 99th percentile (eg, inpatient costs at $6000), set minimum values at $0, and bottom-coded the PCAL outcome at $80, because a practice needs resources to monitor and provide preventive care for even healthy enrollees. When applied, PCAL estimates would be multiplied by the constant needed to ensure budget neutrality.

### PCAL Variables

To align with other MassHealth models, we estimated PCAL following the form of MassHealth’s Social Determinants of Health model that underlies payments to Medicaid ACOs,^11^ with some tailoring (eTable 3 in Supplement 1 defines the variables examined during model development, and eTable 4 in Supplement 1 shows all coefficients for the final PCAL model). Key factors are 2 summary medical acuity scores from Cotiviti, Inc: RxCG, calculated from pharmacy data, and DxCG, calculated from diagnoses.^19^ The models also use indicators for 20 age and sex categories, disability, behavioral health conditions, housing problems, and rurality,^20^ as well as the neighborhood stress score (NSS), a composite measure calculated for Census Block Groups and standardized to have a mean of 0 and SD of 1 in the entire MassHealth population. Higher NSS scores reflect worse socioeconomic conditions. Disability variables, arranged in a dominance hierarchy, are coded as the fraction of the year a member was a client of the Department of Mental Health (DMH), or (if not a DMH client), a client of the Department of Developmental Services, or, if neither a DMH nor Department of Developmental Services client, entitled to Medicaid because of disability. Behavioral health variables were identified from diagnoses: severe mental illness, opioid use disorder, alcohol use disorder, any other substance use disorder, and severe emotional disorder in children. Housing problem indicates either being unhoused (*International Statistical Classification of Diseases, Tenth Revision, Clinical Modification* code Z59.0) or housing instability (3 or more addresses) during 2019. The model also includes 2 interaction terms selected on the basis of a priori concerns, to ensure appropriate payment to subgroups of patients with both social and medical risks that complicate care: housing problem × behavioral health × DxCG and NSS+ × DxCG (where NSS+ is the larger of 0 and NSS). Each specifies larger payment for members of vulnerable groups, calibrated to their medical complexity (DxCG score). For example, PCAL estimates for residents of neighborhoods with worse than average socioeconomic conditions are estimated to cost more on the basis of both their level of neighborhood stress (NSS score) and medical morbidity (DxCG score).

### Statistical Analysis

We calculated the PCAL outcome and used multivariable least square regression to estimate it from member demographics, medical morbidities, and social risks. Because primary care needs do not increase proportionately with patient complexity, we allowed the model to change slope at 5 (RxCG and DxCG) and 20 (DxCG only). For both DxCG and RxCG, less than 4% of scores exceed 5.

Payments based on cost estimates are fair when observed costs (the PCAL outcome) are close to expected costs (the PCAL model estimate). Thus, we examine a model’s performance both through its *R*^2^ and by examining observed-to-expected (O:E) ratios for subgroups of interest to ensure that payments for patients, such as those with multiple medical conditions, behavioral health issues, or housing instability, will be adequate. Ratios greater than 1 indicate underpayment.

We examined PCAL estimates for both patients and practices. In practice-based analyses, we used MassHealth’s records to associate members with their primary care practices, and then sorted patients into deciles of increasing average PCAL-estimated need of the practices in which they were enrolled.

We calculated a validated *R*^2^ for our model using 10-fold cross-validation and 95% CIs for O:Es using the delta method. All statistical analyses were conducted using Stata statistical software version 18.0 (StataCorp).

## Results

The study included 1 014 252 enrolled person-years (mean [SD] age, 25.9 [18.4] years); 538 065 (53.1%) were female, 476 090 (46.9%) were younger than 18 years, 125 438 (12.4%) had some form of disability, 134 993 (13.1%) had a serious mental health diagnosis, 51 494 (5.1%) had an opioid use disorder, and 111 849 (11.0%) had housing problems, including 92 348 person-years (9.1%) with 3 or more distinct addresses and 19 501 person-years (1.9%) with diagnostic code Z59.0 indicating being unhoused (Table 1). Table 2 illustrates the PCAL model. For example, the 2 rows for the RxCG score are interpreted as follows. The model adds 5 × 292 = $1460 when RxCG = 5; yet when RxCG = 6, it only adds $62 more, resulting in a total RxCG increment of $1522. The model separately recognizes costs associated with opioid, alcohol, and other substance use disorders, as well as childhood psychopathology (severe emotional disorder); it sets the NSS coefficient to $5, so that a 1-unit increase in NSS contributes $5 times the DxCG score to the PCAL estimate. Although all other model coefficients were determined empirically, the coefficient for NSS was a policy choice to add resources to practices serving members in resource-poor, historically underserved communities. The PCAL model *R*^2^ was 69.6%, both as fitted and when validated.

**Table 1.** MassHealth Members, Aged 0-64 Years, Enrolled in 3602 Primary Care Practices in 2019^a^

Member characteristics	Person-years, No. (%) (N = 1 014 252)
Age ≤18 y	476 090 (46.9)
Sex	
Female	538 065 (53.1)
Male	476 187 (46.9)
Housing problems^b^	
Unhoused	19 501 (1.9)
Unstably housed (not unhoused)	92 348 (9.1)
Neither of the above	902 403 (89.0)
Disability status^c^	
DMH client	6422 (0.6)
DDS client (not DMH)	18 044 (1.8)
Other disability	100 972 (10.0)
None of the above	888 813 (87.6)
Behavioral health comorbidity	
Serious mental illness	134 993 (13.3)
Opioid use disorder	51 494 (5.1)
Other substance use disorder	44 521 (4.4)
Severe emotional disorder (in children)	35 625 (3.5)
Rural^d^	45 403 (4.5)
Age, mean (SD), y	25.9 (18.4)
Neighborhood Stress score, mean (SD)^e^	0.00 (1.00)
RxCG, mean (SD)^f^	0.99 (2.07)
DxCG, mean (SD)^f^	1.00 (2.09)

Abbreviations: DDS, Department of Developmental Services; DMH, Department of Mental Health.

^a^ Requires 183 or more days of managed care eligibility (MassHealth as primary insurer).

^b^ Housing problem requires either *International Statistical Classification of Diseases, Tenth Revision, Clinical Modification* code Z59.0 (unhoused) or 3 or more addresses during 2019 (unstably housed).

^c^ Disability status indicates MassHealth eligibility as a client of the DMH, or, if not DMH, then as a client of the DDS, or, if neither DMH or DDS, entitled to Medicaid due to disability (other disability).

^d^ MassHealth currently defines rural as rural level 2.^20^

^e^ Neighborhood Stress score is standardized to have a mean of 0 and SD of 1 in the full MassHealth population. Higher scores indicate greater socioeconomic stress.

^f^ RxCG and DxCG (Cotiviti, Inc, version 4.2 models 86 and 88) are risk scores estimating total medical acuity from pharmacy and diagnostic data respectively. Both were normalized to have a mean of 1 in the full MassHealth population, and then top-coded at 30.

**Table 2.** MassHealth Model to Estimate PCAL in 2019 With a Worked Example^a^

Variable	Coefficient	Example^b^	Calculations	Increment, $
Summarized medical morbidity^c^				
RxCG risk score (for each unit, from 0 to 5)	292	RxCG = 9	5 × 292	1460
RxCG (for each unit >5, to a maximum of 30)	62	4	4 × 62	248
DxCG risk score (for each unit, from 0 to 5)	471	DxCG = 10	5 × 471	2355
DxCG (for each unit between 5 and 20)	390	5	5 × 390	1950
DxCG (for each unit >20, to a maximum of 30)	233	0	0	0
Behavioral health				
Serious mental illness	133	0	0	0
Opioid use disorder	407	1	407	407
Other substance use disorder (not opioid or alcohol)	60	0	0	0
Severe emotional distress (in children)	263	0	0	0
Disability (hierarchy)^d^				
Client of the Department of Mental Health	1001	0	0	0
Client of the Department of Developmental Services	661	0	0	0
Medicaid entitlement is due to disability	74	0	0	0
Geography-based measures				
Rural^e^	11	0	0	0
Housing problem × behavioral health × DxCG^f^	12	Housing problem = behavioral health = 1, DxCG = 10	10 × 12	120
NSS+^g^ × DxCG	5	NSS = 3, DxCG = 10	3 × 10 × 5	150
Age-sex categories (20)	Variable	45 y female	149	149
Estimate = sum of (variable values) × (model coefficients)	NA	NA	NA	6839

Abbreviations: NA, not applicable; NSS, Neighborhood Stress Score; PCAL, primary care activity level.

^a^ The 2019 study population includes 1 014 252 MassHealth enrolled person-years, with all variables measured concurrently. Model *R*^2^ is 69.6%. The mean (SD) PCAL is $985 ($1496). In practice, PCAL estimates will be renormalized (multiplied by the appropriate constant) to match a payer-specified total, such as 110% of historical payments to primary care clinicians.

^b^ This example describes a 45-year-old woman with RxCG score = 9, DxCG score = 10, an opioid use disorder diagnosis, and NSS = 3. Since the average value of PCAL in the population is $985, this translates into a relative complexity score of 6.9 (6839/985).

^c^ RxCG and DxCG (Cotiviti, Inc, version 4.2 models 86 and 88) are risk scores estimating total medical acuity from pharmacy and diagnostic data respectively. Both were normalized to have a mean of 1 in the full MassHealth population, and then top-coded at 30. Splines were used to reflect a decreasing influence (of 1 unit increases in either score) on the PCAL outcome.

^d^ Disability variables are coded as fractions of the year members were included in each category (range 0-1).

^e^ MassHealth currently defines rural as rural level 2.^20^

^f^ Housing problem requires either *International Statistical Classification of Diseases, Tenth Revision, Clinical Modification* code Z59.0 (unhoused) or 3 or more addresses during 2019 (unstably housed).

^g^ NSS is standardized to have a mean of 0 and an SD of 1 in the full MassHealth population. Higher scores indicate greater socioeconomic stress. NSS+ recodes negative scores to 0.

The PCAL model estimated well for subpopulations that we commonly examine to ensure model fit (Table 3), with mean expected values within 10% of observed PCAL values for each subgroup shown. Although not explicitly modeled, groups defined by race and ethnicity were estimated well; model-expected costs for 103 388 person-years of Black, non-Hispanic members were 4% higher than observed costs (O:E = 0.96), whereas costs for 349 733 White, non-Hispanic person-years were 1% lower than observed. PCAL estimates were also well matched to observed dollars for groups defined by housing status and neighborhood stress level. Note that the model shifts money (modestly) away from paying for members who live in the most well-resourced neighborhoods, with O:E ratios of 1.01 vs 0.97 for members in the most stressed neighborhoods. The uncertainty in all PCAL-model O:E ratios was less than plus or minus 0.01 (eTable 5 in Supplement 1).

**Table 3.** O:E PCAL Ratios for Select Patient Subgroups^a^^,^^b^

Variable	Person-years	Observed PCAL, $	O:E ratio by model used to estimate expected		
			Average	Age-sex	PCAL
Total	1 014 252	985	1.00	1.00	1.00
Race					
Black, non-Hispanic	103 388	883	0.90	0.93	0.96
Hispanic	78 737	1044	1.06	1.07	1.00
Other (all other nonmissing racial and ethnic categories)	57 008	701	0.71	0.72	0.97
Unknown	425 386	931	0.95	0.99	1.00
White, non-Hispanic	349 733	1114	1.13	1.06	1.01
Neighborhood Stress Score quintile^c^					
Least stressed	202 451	965	0.98	0.95	1.01
Most stressed	204 011	1010	1.03	1.05	0.97
Age, y					
0-6	168 649	752	0.76	1.01	1.01
6-12	162 893	523	0.53	0.99	0.99
13-18	144 548	598	0.61	0.97	1.01
19-26	98 226	846	0.86	0.99	0.99
27-44	241 382	1213	1.23	1.01	1.00
45-64	198 554	1636	1.66	1.00	1.00
Rural^d^	45 403	912	0.93	0.90	1.01
Housing problem^e^					
Unhoused	19 501	3378	3.43	2.75	0.95
Unstably housed (not unhoused)	92 348	1188	1.21	1.30	1.02
Neither	902 403	913	0.93	0.92	1.00
Medical morbidity^f^					
Decile 10 (DxCG >2.33)	101 256	3752	3.81	2.79	0.98
Decile 9	101 629	1742	1.77	1.38	1.06
Deciles 1-8 (DxCG <1.28)	811 366	545	0.55	0.60	1.00

Abbreviations: O:E, observed-to-expected ratio; PCAL, primary care activity level.

^a^ The 2019 study population had 1 092 742 unique MassHealth managed-care-eligible members contributing 1 014 251.6 enrolled person-years distributed across 3602 practices. In practice, PCAL-based payments will be renormalized to match a payer-specified total budget such as 110% of historical payments. eTable 4 in Supplement 1 provides all coefficients for this model.

^b^ All PCAL O:E 95% CIs were narrower than ±0.01, although CIs for other O:E ratios were somewhat larger. To avoid clutter here, eTable 5 in Supplement 1 provides all CIs.

^c^ Neighborhood Stress Score is standardized to have mean of 0 and an SD of 1 in the full MassHealth population. Higher scores indicate greater socioeconomic stress.

^d^ MassHealth currently defines rural as rural level 2.^20^

^e^ Housing problem requires either *International Statistical Classification of Diseases, Tenth Revision, Clinical Modification* code Z59.0 (unhoused) or 3 or more addresses during 2019.

^f^ The DxCG score (Cotiviti, Inc, version 4.2 model 88) estimates total medical acuity from diagnostic data. It was normalized to have mean of 1 in the full MassHealth population and then top-coded at 30.

Table 4 examines practice-level allocations. People are sorted into deciles according to the estimated need of the practices in which they are enrolled. Note that the money paid in 2019 for primary care activities (primary care spend, mean, $580) was less than PCAL (mean, $985) because PCAL also includes dollars from non–primary care spending. Practices in the lowest-need decile mainly treated children (90.8% of individuals were aged 18 years or younger), whereas in the highest decile only 3.2% of individuals were children. Observed mean annual per-member payments (primary care spend) for practices varied widely; practices in the highest decile received nearly 3 times as much per person as those in the lowest decile ($1024 vs $359). The prevalence of each medical and social risk examined in the table increased, often greatly, from the lowest to highest need deciles. For example, just 4.5% of members in decile 1 had a behavioral health diagnosis vs 27.4% in decile 10.

**Table 4.** Characteristics of Patients and Practices and O:E Ratios by Decile of Practice-Level PCAL-Estimated Need^a^

Characteristic	All	Deciles of PCAL-estimated need			
		Lowest		Highest	
		1	2	9	10
Mean annual per person payment for primary care					
Per-person primary care payments in 2019 (primary care spend), $^b^	580	359	426	757	1033
Primary care spend/$580	1.00	0.62	0.74	1.31	1.78
Characteristics of practices and their patients					
Mean practice size	304	129	518	273	131
Age, mean, y	25.9	11.2	13.2	36.6	41.8
Aged ≤18 y, %	46.9	90.8	85.5	15.5	3.2
Female sex, %	53.1	48.8	49.0	55.3	56.0
DxCG, top-coded mean	1.00	0.40	0.51	1.46	2.04
Unstably housed (not unhoused), %	9.8	8.5	9.6	10.0	11.6
Unhoused, %	1.9	0.3	0.4	2.3	5.2
With disability, %	12.4	6.0	7.5	17.4	25.2
With a behavioral health diagnosis, %	13.3	4.5	5.2	20.6	27.4
With any substance use disorder, %	9.9	1.4	1.5	17.7	23.5
Black, %^c^	17.6	13.7	11.2	10.6	17.9
Neighborhood Stress Score, mean	0.00	−0.28	−0.05	−0.12	0.07
O:E with O = mean observed PCAL divided by mean estimated PCAL (E) for 2 models^d^					
E = Age-sex prediction	1.00	0.90	0.95	1.06	1.34
E = PCAL prediction	1.00	1.00	1.01	1.01	1.06

Abbreviations: O:E, observed-to-expected ratio; PCAL, primary care activity level.

^a^ The 2019 study population had 1 092 742 unique MassHealth managed-care-eligible members contributing 1 014 252 enrolled person-years distributed across 3602 practices in 2019. See eTables 2, 3, and 4 in Supplement 1 for PCAL definition details. Each decile has approximately 100 000 members, sorted by increasing value of the mean (per-person) PCAL of the practice in which they are enrolled.

^b^ Primary care spend is less than PCAL, because PCAL also includes some dollars from non–primary-care spending.

^c^ Represented as a fraction of everyone with known race.

^d^ All 95% CIs for these O:E ratios are no wider than ±0.01.

Table 4 also shows how the PCAL model would distribute payments to practices and ACOs compared with payments adjusted for age and sex only. The simpler model would overpay practices in the lowest-need decile by 10% and underpay those in the highest-need decile by 34%; in contrast, the PCAL model would pay the right amount in the lowest need decile and underpay by just 6% for patients in the highest one.

## Discussion

This was a cross-sectional study relating to MassHealth’s 2023 reform that increased primary care spending via capitation, with higher payments for enhanced practice capabilities. Although initial payments are add-ons to historical spending, both ensuring stability and reflecting differences in the apparent needs of patient panels, eventually payments must reflect the needs of contemporaneous patients to support practices caring for complex patients.

Unlike the fee-for-service payment system it replaces, prospective payment is agnostic to how practices mix traditional visits and other forms of interactions with patients, aligning with recommendations by the National Academies for Science, Engineering, and Medicine and others.^21,22^ Massachusetts Senate Bill S750, introduced in 2023, proposes similar reforms for commercial payers.^23^

To partially address concerns that aggressive diagnostic coding inflates the appearance of need, we used pharmacy data (in addition to medical diagnoses) to characterize risk, recognized both individual and neighborhood-level measures of social risk, and estimated lower increases in service use for increments in medical risk scores that are already high. Another concern is that risk models can enshrine existing unfair care patterns, for example, by underpaying for minoritized populations.^24^ Thus, for example, we examined racial subgroups to confirm that the PCAL model pays a bit more for minoritized populations than their practitioners currently receive. We started modeling with the exact variables in MassHealth’s global payment model (eTable 4 in Supplement 1), using an iterative process whose key goal was to not underpay for any subgroup of people thought likely to need extra attention.

Public payers should look for ways to improve payment equity, as MassHealth did by imposing a coefficient plus $5 on the NSS,^12^ a value judged large enough to make a difference for practices operating in poor neighborhoods and verified to be small enough to avoid creating substantial distortions in other model coefficients. Bergquist and colleagues^25^ have proposed a clever alternative way to build models incorporating desired policy goals. We used MassHealth’s NSS in the PCAL model to recognize differences in neighborhood resources. Going forward, MassHealth is considering a separate risk equity payment indexed directly to a social risk measure, rather than including such a variable in risk modeling. Although the Area Deprivation Index is commonly used by federal and state programs for this purpose, it has a serious methodologic flaw,^26^ and other area-based indices correlate better with population health.^27^

### Limitations

Limitations of this study include (1) not directly measuring the effort required to deal comprehensively with patients’ primary care needs, (2) not capturing key aspects of patient situations (eg, family circumstances and criminal justice involvement) that underlie need, and (3) not specifying quality measures for identifying and rewarding exemplary practices. We used 2019 data to avoid COVID-19 disruptions. Future models should reflect the new normal, including the effects of COVID-19 and its sequelae.

Our model is based on MassHealth’s unique data, policy priorities, and desire for alignment with its other models. Others should build and test their own models.

The decision to increase total spending on primary care is foundational; PCAL is used to allocate a predetermined total budget among practices. Future work should consider tailored payments for distinct kinds of primary care practices (eg, pediatric, family medicine, and general internal medicine) to address, for example, concerns that existing spending may reflect underinvestment in children’s health.

## Conclusions

MassHealth’s 2023 reform encouraged and supported primary care clinicians to organize themselves in multidisciplinary teams to provide integrated and comprehensive primary care. This cross-sectional study has shown how risk models can be used to develop prospective payments that support caring for complex patients.

## Notes

Supplement 1. **eTable 1.** Enhanced Delivery Expectations for Primary Care Practice Tiers in the MassHealth Sub-capitation Program **eTable 2.** Defining PCAL and Other Modeling Decisions **eTable 3.** Variables Examined in Model Development **eTable 4.** Coefficients for Three Models Predicting PCAL **eTable 5.** 95% Confidence Intervals for Table 3 Observed-to-Expected (O:E) Ratios

Supplement 2.
Data Sharing Statement

## References

[1] Robinson SK, Meisnere M, Phillips RL Jr, McCauley L, eds. Implementing High-Quality Primary Care: Rebuilding the Foundation of Health Care. National Academies of Sciences, Engineering, and Medicine; 2021. 34251766

[2] Basu S, Berkowitz SA, Phillips RL, Bitton A, Landon BE, Phillips RS. Association of primary care physician supply with population mortality in the United States, 2005-2015. JAMA Intern Med. 2019;179(4):506-514. doi:10.1001/jamainternmed.2018.7624 30776056 PMC6450307

[3] Sandvik H, Hetlevik Ø, Blinkenberg J, Hunskaar S. Continuity in general practice as predictor of mortality, acute hospitalisation, and use of out-of-hours care: a registry-based observational study in Norway. Br J Gen Pract. 2022;72(715):e84-e90. doi:10.3399/BJGP.2021.0340 34607797 PMC8510690

[4] Jabbarpour Y, Jetty A, Byun H, Siddiqi A, Petterson S, Park J. The health of US primary care: 2024 scorecard report—no one can see you now; five reasons why access to primary care is getting worse (and what needs to change). The Milbank Memorial Fund and The Physicians Foundation. February 28, 2024. Accessed December 20, 2024. https://www.graham-center.org/content/dam/rgc/documents/publications-reports/reports/2024-scorecard-final-report.pdf

[5] Koller CF, Betancourt JR, Miller ME. Out of balance: fixing our health system’s neglect of primary care. Health Affairs Forefront. September 5, 2024. Accessed December 20, 2024. https://www.healthaffairs.org/content/forefront/out-balance-fixing-our-health-system-s-neglect-primary-care

[6] Massachusetts Health Policy Commission. ACO Policy brief—transforming care: an introduction to accountable care organizations in Massachusetts. April 1, 2018. Accessed August 27, 2024. https://masshpc.gov/sites/default/files/2023-01/ACO%20brief%20%231.pdf

[7] Nelson DB, Schwarz R, Dar M. Primary care sub-capitation in Medicaid: improving care delivery in the safety net. J Gen Intern Med. 2023;38(5):1288-1290. doi:10.1007/s11606-023-08063-0 36750508 PMC9904520

[8] Milbank Memorial Fund. How Massachusetts Medicaid is paying for primary care teams to take care of people, not doctors to deliver services. August 15, 2023. Accessed August 27, 2024. https://www.milbank.org/news/how-massachusetts-medicaid-is-paying-for-primary-care-teams-to-take-care-of-people-not-doctors-to-deliver-services/

[9] Massachusetts Executive Office of Health and Human Services. MassHealth primary care sub-capitation: care delivery transformation. 2024. Accessed August 27, 2024. https://www.mass.gov/info-details/masshealth-primary-care-sub-capitation-care-delivery-transformation#intro-to-clinical-tiers

[10] Jaffery J, Safran D. Addressing social risk factors in value-based payment: adjusting payment not performance to optimize outcomes and fairness. Health Affairs Forefront. April 19, 2021. Accessed December 20, 2024. https://www.healthaffairs.org/content/forefront/addressing-social-risk-factors-value-based-payment-adjusting-payment-not-performance

[11] Alcusky MJ, Mick EO, Allison JJ, . Paying for medical and social complexity in Massachusetts Medicaid. JAMA Netw Open. 2023;6(9):e2332173. doi:10.1001/jamanetworkopen.2023.32173 37669052 PMC10481227

[12] Sabatino M, Alcusky MJ, Mick EO, Ash AS. Risk adjustment and health equity. Health Aff (Millwood). 2023;42(5):732. doi:10.1377/hlthaff.2023.00218 37126748

[13] Centers for Medicare & Medicaid Services. Risk adjustment. 2024. Accessed August 27, 2024. https://www.cms.gov/medicare/payment/medicare-advantage-rates-statistics/risk-adjustment

[14] Pope GC, Kautter J, Ellis RP, . Risk adjustment of Medicare capitation payments using the CMS-HCC model. Health Care Financ Rev. 2004;25(4):119-141. 15493448 PMC4194896

[15] Centers for Medicare & Medicaid Services. Making Care Primary (MCP) Model. 2023. Accessed December 7, 2023. https://www.cms.gov/priorities/innovation/innovation-models/making-care-primary

[16] Maryland Department of Health. Maryland Primary Care Program: HEART Payment Playbook. August 2024. Accessed August 27, 2024. https://health.maryland.gov/mdpcp/Documents/MDPCP_HEART_Payment_Playbook.pdf

[17] Centers for Medicare & Medicaid Services. Guiding an improved dementia experience (GUIDE) model participant incentives to participate factsheet. 2024. Accessed November 19, 2024. https://www.cms.gov/files/document/guide-participant-model-incentives-factsheet.pdf

[18] Ash AS, Ellis RP. Risk-adjusted payment and performance assessment for primary care. Med Care. 2012;50(8):643-653. doi:10.1097/MLR.0b013e3182549c74 22525609 PMC3394905

[19] Cotiviti, Inc. DxCG Intelligence Bundle & Model Directory and Science Guide, version 5.3.1. 2019. Accessed October 23, 2023. https://training.dxs-systems.com/wp-content/uploads/2021/09/010DXS-Release-Notes-Version-5.3.1-General-Release.pdf

[20] MA State Office of Rural Health. Rural definition. Accessed December 19, 2024. https://www.mass.gov/doc/rural-definition-detail-0/download

[21] Phillips RL Jr, McCauley LA, Koller CF. Implementing high-quality primary care: a report from the National Academies of Sciences, Engineering, and Medicine. JAMA. 2021;325(24):2437-2438. doi:10.1001/jama.2021.7430 33944903

[22] Basu S, Phillips RS, Song Z, Bitton A, Landon BE. High levels of capitation payments needed to shift primary care toward proactive team and nonvisit care. Health Aff (Millwood). 2017;36(9):1599-1605. doi:10.1377/hlthaff.2017.0367 28874487

[23] The 193rd General Court of the Commonwealth of Massachusetts. An act relative to primary care for you. Bill S.750. 2024. Accessed August 27, 2024. https://malegislature.gov/Bills/193/SD2233

[24] McWilliams JM, Weinreb G, Ding L, Ndumele CD, Wallace J. Risk adjustment and promoting health equity in population-based payment: concepts and evidence. Health Aff (Millwood). 2023;42(1):105-114. doi:10.1377/hlthaff.2022.00916 36623215 PMC9901844

[25] Bergquist SL, Layton TJ, McGuire TG, Rose S. Data transformations to improve the performance of health plan payment methods. J Health Econ. 2019;66:195-207. doi:10.1016/j.jhealeco.2019.05.005 31255968 PMC7442111

[26] Hannan EL, Wu Y, Cozzens K, Anderson B. The neighborhood atlas area deprivation index for measuring socioeconomic status: an overemphasis on home value. Health Aff (Millwood). 2023;42(5):702-709. doi:10.1377/hlthaff.2022.01406 37126749

[27] Dyer Z, Alcusky MJ, Galea S, Ash A. Measuring the enduring imprint of structural racism on American neighborhoods. Health Aff (Millwood). 2023;42(10):1374-1382. doi:10.1377/hlthaff.2023.00659 37782878 PMC10804769

