# Supplementary material for: Supporting Primary Care for Medically and Socially Complex Patients in Medicaid Managed Care
Source: JAMA Netw Open. 2025 Feb 3;8(2):e2458170. doi: 10.1001/jamanetworkopen.2024.58170 (PMC11791707; doi:10.1001/jamanetworkopen.2024.58170)
Supplement: Supplement 1. — eTable 1. Enhanced Delivery Expectations for Primary Care Practice Tiers in the MassHealth Sub-capitation Program eTable 2. Defining PCAL and Other Modeling Decisions eTable 3. Variables Examined in Model Development eTable 4. Coefficients for Three Models Predicting PCAL eTable 5. 95% Confidence Intervals for Table 3 Observed-to-Expected (O:E) Ratios [file jamanetwopen-e2458170-s001.pdf]

Supplemental Online Content

Ash AS, Alcusky MJ, Ellis RP, Sabatino MJ, Eanet FE, Mick EO. Strengthening primary care in Medicaid managed care. *JAMA Netw Open*. 2025;8(2):e2458170. doi:10.1001/jamanetworkopen.2024.58170

eTable 1. Enhanced Delivery Expectations for Primary Care Practice Tiers in the MassHealth Sub-capitation Program
eTable 2. Defining PCAL and Other Modeling Decisions
eTable 3. Variables Examined in Model Development
eTable 4. Coefficients for Three Models Predicting PCAL
eTable 5. 95% Confidence Intervals for Table 3 Observed-to-Expected (O:E) Ratios

This supplemental material has been provided by the authors to give readers additional information about their work.

© 2025 Ash AS et al. *JAMA Network Open*.

eTable 1: Enhanced Delivery Expectations for Primary Care Practice Tiers in the MassHealth Sub-capitation Program
As circulated by MassHealth, this table outlines the care model criteria for primary care practices participating in a MassHealth ACO to qualify for each Tier of the Primary Care Sub-capitation program.*
1. Practices with Tier 1 designation must fulfill **all** Tier 1 care model requirements<br>2. Practices with Tier 2 designation must fulfill **all** Tier 1 and Tier 2 care model requirements<br>3. Practices with Tier 3 designation must fulfill **all** Tier 1, 2, and 3 care model requirements
**Tier 1 Care Model Criteria**<br>(estimated add-on $4.00-6.00 per member per month)<br>*Practices must meet the following criteria:*
**A. Care Delivery**
Traditional primary care services
Referral to specialty care services
Oral health screening and referral for treatment
Mental health and substance use disorder screening with referral and treatment
Bi-directional behavioral health referrals with monitoring
In coordination with the ACO, written processes and procedures in place to ensure baseline care coordination is provided to all members
**B. Structure and Staffing**
Same-day urgent care access
24/7 clinical advice & support line
Telehealth capable
Upon participating in the sub-capitation program, the practice should see no reduction in accessible hours
**C. Population Specific Expectations**
Only applicable to practices serving members under age 21
Coordination with services associated with the Children's Behavioral Health Initiative (CBHI)
Coordination with Massachusetts Child Psychiatry Access Program services
Postpartum depression maternal screening
Developmental and behavioral health screenings
Fluoride varnish for patients ages 1-5
Only applicable to practices serving adults:

© 2025 Ash AS et al. *JAMA Network Open*.

All providers on-site are waivered and trained to prescribe medication-assisted treatment
**<u>Tier 2 Care Model Criteria</u>**<br>(estimated add-on $6.00-8.00 per member per month)<br>*In addition to all Tier 1 requirements, practices must meet the following criteria*
**A. Care Delivery**<br>Capability to provide brief interventions for behavioral health<br>Referral partners with tele-behavioral health capability<br>**B. Structure and Staffing**<br>E-consultations are available in 3 or more specialties<br>Open for at least 1 session on evenings or weekends<br>Maintain at least 1 team-based staff role on-site (CHW, Peer, MSW, LICSW, RN case manager)<br>Maintain a consulting behavioral health clinician<br>**C. Population Specific Expectations**<br>*Only applicable to practices serving members under age 21*<br>On-site children, youth and family (CYF)-specific expertise staff at least part-time<br>Practice-based staff point of coordination (POC) for CBHI services and family resource centers (FRC)<br>All providers on-site are waivered and trained to prescribe medication-assisted treatment<br>*Only applicable to practices serving adults:*<br>Ability to provide more than 1 type of long-acting reversible contraception on-site<br>Capacity to provide medication-assisted treatment on-site
**<u>Tier 3 Care Model Criteria</u>**<br>(estimated add-on $11.00-13.00 per member per month)<br>*In addition to all Tier 1 and Tier 2 requirements, practices must meet the following criteria:*
**A. Care Delivery**<br>Clinical pharmacist visits **<u>or</u>** at least one type of group visits (e.g., behavioral health, substance use disorder treatment, antenatal care)<br>**B. Structure and Staffing**<br>E-consultations are available in 5 or more specialties<br>Open for at least 3 sessions in evenings or weekends (at least one in-person)<br>Maintain at least 3 team-based staff roles on-site (CHW, Peer, MSW, LICSW, RN case manager)<br>Maintain an independently licensed behavioral health clinician and prescriber on-site part-time<br>**C. Population-Specific Expectations**<br>*Only applicable to practices serving members under age 21*

© 2025 Ash AS et al. *JAMA Network Open*.

On-site children, youth and family-specific expertise staff full-time
Ability to provide more than 1 type of long-acting reversible contraception on-site
Medication-assisted treatment maintenance therapy is available on-site for patients 12 years of age and older
Only applicable to practices serving adults:
Intrauterine device (IUD) and contraceptive implant available on-site
Capability for next-business-day medication-assisted treatment induction and follow-up

* Nelson DB, Schwarz R, Dar M. Primary Care Sub-capitation in Medicaid: Improving Care Delivery in the Safety Net. *J Gen Intern Med*. Apr 2023;38(5):1288-1290. doi:10.1007/s11606-023-08063-0

© 2025 Ash AS et al. *JAMA Network Open*.

eTable 2: Defining PCAL and Other Modeling Decisions			
**Original (2012) and Updated (2023) MassHealth Models**			
**Activity Categories whose costs contribute to PCAL***	**Original PCAL**<br>**% of All Such Costs Contributing**	**New PCAL**<br>**% of All Such Costs Contributing**	**New PCAL top-coding****
Primary Care core and "semi-core"	98%	100%	$10,000
Specialty care-related	6%	6%	$50,000
Hospital care-related	6%	6%	$100,000
Emergency Department visits	17%	30%	$3,000
Prescription drug use	12%	9%	$40,000

*We used BETOS (type of service) codes to classify all claims into one of the above categories. Contact the authors for more details if you would like to replicate our mapping.
**Set at the ~99th percentile of prescription Rx use

Modeling Decisions

- The PCAL outcome was first constructed as the sum of 100% of all primary care core costs + 6% of each of specialty care and hospital costs + 17% of ED costs + 12% of prescription drug costs. This outcome was then annualized (that is, divided by W = fraction of the year enrolled) and all analyses were weighted by W. For example, an observation for a person enrolled for half the year with observed PCAL = $1000 contributed ½ of a person-year, with an annualized PCAL of $2000/year.
- Both RxCG and DxCG morbidity scores were first standardized to have mean = 0 and SD = 1 in the full MassHealth managed-care-eligible population. In modeling, each score was top-coded at 30.
- After fitting the model (and before examining O:E ratios), model predictions were bottom-coded at $80 (to ensure a minimal payment for monitoring the care of even the heathiest patients), and the coefficient for NSS+ was set to be positive $5, rather than allowing it to be negative $2, as the modeling algorithm would have assigned it. This choice was informed by an equity-based policy decision to spend more money on patients living in less-well-resourced areas, and on analyses showing that $5 was large enough to make a meaningful difference to practices, but not so large as to substantially distort other coefficients or reduce the model's explanatory power.
- We increased the contribution of ED visit costs to new PCAL (from 17% to 30%), since ED use is more common in a Medicaid population than in a commercially insured one, taking up more of a doctor's time.
- Note that higher PCAL payments are not tied to a patient's greater use of the ED – or to any other service use – but rather to patient characteristics that contribute to a higher predicted PCAL.

© 2025 Ash AS et al. *JAMA Network Open*.

eTable 3: Variables Examined in Model Development		
**Behavioral Health and Substance Abuse (by presence of ICD-10-CM codes)**	Severe mental illness (SMI)	DxCG v6.0 DxGroups: 403, 403, 405, 408, 410
	Opioid use disorder (OUD)	F11.10, F11.120, F11.121, F11.122, F11.129, F11.13, F11.14, F11.150, F11.151, F11.159, F11.181, F11.182, F11.188, F11.19, F11.20, F11.220, F11.221, F11.222, F11.229, F11.23, F11.24, F11.250, F11.251, F11.259, F11.281, F11.282, F11.288, F11.29
	Alcohol use disorder (AUD)	F10.10, F10.12x, F10.13x, F10.14, F10.15x, F10.18x, F10.19, F10.20, F10.22x, F10.23x, F10.24, F10.25x, F1026, F10.27, F10.28x, F10.29. Note that AUD is not used in the final PCAL model.
	Other substance use disorder (OTHER_SUD)	F12.10, F12.12x, F12.13, F12.50x, F12.18, F12.188, F12.19, F12.20, F12.22x, F12.23, F12.25x, F12.29, F13.10, F13.12x, F13.13x, F13.14, F13.15x, F13.18x, F13.19, F13.20, F13.22x, F13.23x, F13,24, F13.25x, F13.26, F13.27, F13.28x, F13.29, F14.10, F14.12x, F14.13, F14.14, F14.15x, F14.18x, F14.19, F14.20, F14.22x, F14.23x, F14,24, F14.25x, F14.28x, F14.29, F15.10, F15.12x, F15.13, F15.14, F15.15x, F15.18x, F15.19, F15.20, F15.22x, F15.23x, F15,24, F15.25x, F15.28x, F15.29, F16.10, F16.12x, F16.14, F16.15x, F16.18x, F16.19, F16.20, F16.22x, F16,24, F16.25x, F16.28x, F14.69, F18.10, F18.12x, F18.14, F18.15x, ,F18.17, F18.18x, F18.19, F18.20, F18.22x, F18.24, F18.25x, F18.27, F18.28x, F18.29, , F19.10, F19.12x, F19.13x, F19.14, F19.15x, F19.16, F19.17, F19.18x, F19.19, F19.20, F19.22x, F19.23x, F19,24, F19.25x, F19.26, F19.27, F19.28x, F19.29
	Severe emotional disorder (SED), only coded in children	Personality disorders, eating disorders, mood and anxiety disorders, suicide attempts, and psychoses defined by DxCG v6.0 DxGroups 391, 392, 402 – 411, 417, 418, 420, but not separately-identified psychosis diagnoses (F20.x, F21.x, F22.x, F23.x, F24.x, F25.x, F30.2, F31.2, F31.5, F32.3, F33.3)
	Behavioral Health (BH)	Presence of any of the above
	Homeless	Presence of ICD-10-CM code Z59.0x

© 2025 Ash AS et al. *JAMA Network Open*.

**Housing Problem**	Unstably Housed	Having 3 or more addresses in 2019
	Housing Problem (HP)	Either Unstably Housed or Homeless
**Rurality**	Rural	Rurality indicator, defined for this model from https://geo-massdot.opendata.arcgis.com/datasets/urban-boundaries-2010. November 30, 2021. Accessed on February 20, 2024. Going forward, MassHealth plans to define "Rural" as level 2 rurality from the Massachusetts State Office of Rural Health. https://www.mass.gov/doc/rural-definition-detail-0/download. Accessed February 20, 2024.
**Medical Morbidity**	RxCG score	Both RxCG and DxCG morbidity scores were first standardized to have mean = 0 and SD = 1 in the full MassHealth managed-care-eligible population. For use in modeling, each score was top-coded at 30, and spline knots were introduced at 5 (for both scores) and 20 (DxCG only).
	DxCG score	
**Disability Hierarchy**	DMH	DMH = Client of the Department of Mental Health; DDS = Client of the Department of Developmental Services (but only if not DMH); Other Disability, if neither DMH nor DDS, but "disability" is the reason for MassHealth entitlement;. DMH_PCT, DDS_PCT, and Other DISAB_PCT, the proportions of the year that a member was associated with each of these categories, were used in modeling.
	DDS	
	Other Disability	
Neighborhood Stress Score	NSS	A census-block-group level composite measure based on 7 American Community Survey variables (defined in Ash AS, Mick EO, Ellis RP, Kiefe CI, Allison J, Clark MA. Social determinants of health in managed care payment formulas. *JAMA Intern Med*. 2017;177(10):1424-1430. doi:10.1001/jamainternmed.2017.3317). NSS is standardized to have mean = 0 and SD = 1 in the entire MassHealth population; NSS+ = max (NSS, 0).
Age/Sex Indicators	FEM*** and MALE***	Where *** indicates age in years in the following categories: 0-1; 2-5,6-12, 13-17, 18-24, 25-34, 35-44, 45-54, 55-59, 60-64.

© 2025 Ash AS et al. *JAMA Network Open*.

eTable 4: Coefficients for Three Models Predicting PCAL

PCAL Model* (R^2 = 69.6)

Variable	Coefficien t	t
constant	272	77.8
FEM: 0 and 1 y	237	34.0
FEM: 2 to 5 y		REF
FEM: 6 to 12 y	-19	-4.4
FEM: 13 to 17 y	-63	-13.2
FEM: 18 to 24 y	-12	-2.3
FEM: 25 to 34 y	-47	-10.7
FEM: 35 to 44 y	-82	-17.4
FEM: 45 to 54 y	-79	-15.7
FEM: 55 to 59 y	-84	-13.9
FEM: 60 to 64 y	-123	-19.9
MALE: 0 and 1 y	248	36.0
MALE: 2 to 5 y	11	2.3
MALE: 6 to 12 y	-8	-1.8
MALE: 13 to 17 y	-108	-22.8
MALE: 18 to 24 y	-193	-37.6
MALE: 25 to 34 y	-181	-37.6
MALE: 35 to 44 y	-174	-34.1
MALE: 45 to 54 y	-243	-46.8
MALE: 55 to 59 y	-279	-44.6
MALE: 60 to 64 y	-282	-42.7
RxCG	292	299.3
RxCG, when>5	-230	-164.2

Starting PCAL Model* (R^2 = 68.1)

Variable	Coefficien t	t
constant	342	95.9
FEM: 0 and 1 y	322	45.2
FEM: 2 to 5 y		REF
FEM: 6 to 12 y	-37	-8.4
FEM: 13 to 17 y	-39	-7.9
FEM: 18 to 24 y	33	6.5
FEM: 25 to 34 y	16	3.5
FEM: 35 to 44 y	0	0.0
FEM: 45 to 54 y	20	3.9
FEM: 55 to 59 y	28	4.5
FEM: 60 to 64 y	8	1.2
MALE: 0 and 1 y	351	49.8
MALE: 2 to 5 y	26	5.3
MALE: 6 to 12 y	-16	-3.7
MALE: 13 to 17 y	-104	-21.6
MALE: 18 to 24 y	-202	-38.5
MALE: 25 to 34 y	-190	-38.5
MALE: 35 to 44 y	-153	-29.2
MALE: 45 to 54 y	-174	-32.8
MALE: 55 to 59 y	-176	-27.5
MALE: 60 to 64 y	-163	-24.2
RxCG	161	299.9

Age-Sex model (R^2 = 8.4%)

Variable	Coefficient	t
constant	677	56.0
FEM: 0 and 1 y	-75	-9.9
FEM: 2 to 5 y		REF
FEM: 6 to 12 years	71	8.6
FEM: 6 to 12 y	384	44.3
FEM: 13 to 17 y	655	86.5
FEM: 18 to 24 y	788	98.5
FEM: 25 to 34 y	1044	123.4
FEM: 35 to 44 y	1203	117.5
FEM: 45 to 54 y	1172	112.0
FEM: 55 to 59 y	830	69.7
FEM: 60 to 64 y	98	11.6
MALE: 0 and 1 y	6	0.8
MALE: 2 to 5 y	-11	-1.4
MALE: 6 to 12 y	-3	-0.3
MALE: 13 to 17 y	392	47.5
MALE: 18 to 24 y	655	75.1
MALE: 25 to 34 y	896	101.2
MALE: 45 to 54 y	1125	105.4
MALE: 55 to 59 y	1191	105.9
MALE: 60 to 64 y	562	93.2

© 2025 Ash AS et al. *JAMA Network Open*.

DxCG	471	429.0	DxCG	419	679.4
DxCG, when>5	-81	-48.2			
DxCG, when>20	-157	-37.3			
SMI	133	46.3	SMI only	322	113.6
OUD	407	94.0	OUD only	578	111.3
OTHER_SUD	60	12.8	SMI+OUD	843	143.3
SED	263	55.8			
DMH_PCT	1001	95.6	DMH_PCT	1118	104.3
DDS_PCT	661	106.0	DDS_PCT	745	116.9
Oth DISAB_PCT	74	24.0	Other DISAB_PCT	142	45.0
NSS+_x_DxCG	-2	-8.2	NSS+_x_DxCG	-3	-12.5
RURAL	11	3.0	RURAL	1	0.2
BH_x_HP_x_DxCG	12	29.1	BH_x_HP_x_DxCG	9	21.4

- The “Starting PCAL” model used the exact same predictors that MassHealth was using to allocate global payments among the ACOs; the (final) PCAL model was modified to better predict the PCAL outcome.

Notes:

- The study population includes 1,014,625 MassHealth enrolled person-years.
- RxCG and DxCG (Cotiviti, Inc.’s v4.2 Models 86 and 88) are risk scores predicting total medical acuity from pharmacy and diagnostic data, respectively. Both were normalized to have mean = 1 in the full MassHealth population, and then top-coded at 30. Spline knots were used at 5 (RxCG and DxCG) and 20 (DxCG only) to accommodate non-linearity relationships between these scores and the PCAL outcome.
- OTHER SUD: Substance Use Disorder other than alcohol or opioids.
- Disability variables are coded as a fraction of the year members were included in each category (range 0-1).
- NSS is a neighborhood stress score with mean=0 and SD=1; NSS+ has recoded negative scores to 0.
- Rural is an indicator for rurality, originally defined using the MassDOT definition (Massachusetts Department of Transportation. Urban Boundaries 2010. https://geo-massdot.opendata.arcgis.com/datasets/urban-boundaries-2010. November 30, 2021. Accessed on February 20, 2024). Going forward, MassHealth will be defining rural as level 2 rurality (Rural Definition. Massachusetts State Office of Rural Health. Accessed February 20, 2024. https://www.mass.gov/doc/rural-definition-detail-0/download).
- The “Housing Problem” indicator is switched on for either homelessness (ICD-10-CM code Z59.0) or unstable housing (>3 addresses in one year). It is then multiplied by the DxCG score and an indicator for having any behavioral disorder.
- Average PCAL is $985. In practice, PCAL-based payments would be re-normalized (multiplied by a constant) to match the average amount the payer specifies, for example, 110% of historical payments.

© 2025 Ash AS et al. *JAMA Network Open*.

eTable 5. 95% Confidence Intervals for Table 3 Observed-to-Expected (O:E) Ratios[a]						
				O:E ratio with E predicted by		
		Observed PCAL ($)		Average	Age-Sex Model	PCAL Model
	Person Years	Mean	[95% CI]	O:E [95% CI]	O:E [95% CI]	O:E [95% CI]
**Total**	1,014,252	$985	[982 ,988]	1.00 [1.00, 1.00]	1.00 [1.00, 1.00]	1.00 [1.00, 1.00]
**Race**						
Black, Non-Hispanic	103,388	$883	[875 ,891]	0.90 [0.89, 0.90]	0.93 [0.92, 0.94]	0.96 [0.96, 0.97]
Hispanic	78,737	$1044	[1033, 1054]	1.06 [1.05, 1.07]	1.07 [1.06, 1.08]	1.00 [0.99 ,1.00]
Other	57,008	$701	[692, 709]	0.71 [0.70, 0.72]	0.72 [0.71, 0.73]	0.97 [0.96, 0.98]
Unknown	425,386	$931	[927, 935]	0.95 [0.94, 0.95]	0.99 [0.99, 0.99]	1.00 [1.00, 1.00]
White, Non-Hispanic	349,733	$1114	[1108, 1119]	1.13 [1.13, 1.14]	1.06 [1.05, 1.06]	1.01 [1.01, 1.02]
**Neighborhood Stress (NSS)[b] Quintile**						
Least Stressed	202,451	$965	[959, 971]	0.98 [0.97, 0.99]	0.95 [0.94, 0.95]	1.01 [1.01, 1.02]
Most Stressed	204,011	$1010	[1004, 1017]	1.03 [1.02, 1.03]	1.05 [1.04, 1.06]	0.97 [0.97, 0.97]
**Age in years**						
0-6	168,649	$752	[748, 757]	0.76 [0.76, 0.77]	1.01 [1.00, 1.01]	1.01 [1.01, 1.02]
6-12	162,893	$523	[519, 526]	0.53 [0.53, 0.53]	0.99 [0.98, 1.00]	0.99 [0.98, 0.99]
13-18	144,548	$598	[594, 603]	0.61 [0.60, 0.61]	0.97 [0.97, 0.98]	1.01 [1.00, 1.01]
19-26	98,226	$846	[838, 853]	0.86 [0.85, 0.87]	0.99 [0.98, 1.00]	0.99 [0.99, 1.00]
27-44	241,382	$1213	[1206, 1219]	1.23 [1.22, 1.24]	1.01 [1.01, 1.02]	1.00 [1.00, 1.00]
45-64	198,554	$1636	[1627, 1645]	1.66 [1.65, 1.67]	1.00 [0.99, 1.01]	1.00 [1.00, 1.00]
**Rural[c]**	45,403	$912	[900, 924]	0.93 [0.91, 0.94]	0.90 [0.89, 0.91]	1.01 [1.00, 1.01]
**Housing Problems[d]**						
Homeless	19,501	$3378	[3333, 3422]	3.43 [3.38, 3.47]	2.75 [2.72, 2.78]	0.95 [0.94, 0.96]
Unstably Housed	92,348	$1188	[1178, 1199]	1.21 [1.20, 1.22]	1.30 [1.29, 1.31]	1.02 [1.01, 1.02]
Neither	902,403	$913	[910, 915]	0.93 [0.92, 0.93]	0.92 [0.92, 0.93]	1.00 [1.00, 1.00]
**Morbidity Deciles[e]**						
D 10 (DxCG>2.33)	101,256	$3752	[3735, 3769]	3.81 [3.79, 3.83]	2.79 [2.77, 2.80]	0.98 [0.98, 0.98]

© 2025 Ash AS et al. *JAMA Network Open*.

D 9	101,629	$1742	[1734, 1749]	1.77 [1.76, 1.78]	1.38 [1.37, 1.38]	1.06 [1.05, 1.06]
D 1-8 (DxCG<1.28)	811,366	$545	[544, 546]	0.55 [0.55, 0.55]	0.60 [0.60, 0.60]	1.00 [0.99, 1.00]

[a] The 2019 study population had 1,092,742 unique MassHealth managed-care-eligible members contributing 1,014,251.6 enrolled person-years distributed across 3602 practices. In practice, PCAL-based payments will be re-normalized to match a payer-specified total budget such as 110% of historical payments. eTable 4 provides all coefficients for this model. Confidence intervals were computed using the delta method.

[b] Neighborhood Stress Score (NSS) is standardized to have mean = 0 and SD = 1 in the full MassHealth population. Higher scores indicate greater socioeconomic stress.

[c] MassHealth currently defines rural as “Rural Level 2” from https://www.mass.gov/doc/rural-definition-detail-0/download

[d] Housing problem requires either ICD-10 code Z59.0 (unhoused) or 3 or more addresses during 2019. Only those who are not unhoused are counted in the unstably housed row.

[e] The DxCG score (Cotiviti, Inc.’s v4.2 Model 88) predicts total medical acuity from diagnostic data. It was normalized to have mean = 1 in the full MassHealth population and then top-coded at 30.

© 2025 Ash AS et al. *JAMA Network Open*.
