# Supplementary material for: Supporting Primary Care for Medically and Socially Complex Patients in Medicaid Managed Care
Source: JAMA Netw Open. 2025 Feb 3;8(2):e2458170. doi: 10.1001/jamanetworkopen.2024.58170 (PMC11791707; doi:10.1001/jamanetworkopen.2024.58170)
Supplement: Supplement 2. — Data Sharing Statement [file jamanetwopen-e2458170-s002.pdf]

Data Sharing Statement

Ash. Strengthening Primary Care in Medicaid Managed Care. *JAMA Netw Open*. Published February 03, 2025. doi:10.1001/jamanetworkopen.2024.58170

Data
Data available: No

Additional Information
Explanation for why data not available: The data belong to Mass Health; they are not ours to share. Any legitimate researcher can request these data from the owner directly.
